# Assessing the safety attitudes questionnaire (SAQ), German language version in Swiss university hospitals - a validation study

**DOI:** 10.1186/1472-6963-13-347

**Published:** 2013-09-10

**Authors:** Natalie Zimmermann, Kaspar Küng, Susan M Sereika, Sandra Engberg, Bryan Sexton, René Schwendimann

**Affiliations:** 1 Institute of Nursing Science, University of Basel, Bernoullistrasse 28, Basel 4056, Switzerland; 2 Department of Orthopedic Surgery, University Hospital of Bern, Freiburgstrasse 1, Bern 3000, Switzerland; 3 Department of Cardiovascular Surgery, University Hospital of Bern, Bern 3000, Switzerland; 4 Department of Health & Community Systems and Biostatistics, School of Nursing and Graduate School of Public Health, University of Pittsburgh, 360 Victoria Building, 3500 Victoria Street, Pittsburgh, PA 15261, USA; 5 Department of Health Promotion & Development, School of Nursing, University of Pittsburgh, 440 Victoria Building, 3500 Victoria Street, Pittsburgh, PA 15261, USA; 6 Department of Psychiatry, School of Medicine, Duke University, Durham, NC 27715, USA

**Keywords:** Safety attitudes questionnaire (SAQ), Patient safety, Psychometrics, Swiss hospital setting

## Abstract

**Background:**

Improving patient safety has become a major focus of clinical care and research over the past two decades. An institution’s patient safety climate represents an essential component of ensuring a safe environment and thereby can be vital to the prevention of adverse events. Covering six patient safety related factors, the Safety Attitudes Questionnaire (SAQ) is a validated and widely used instrument to measure the patient safety climate in clinical areas. The objective of this study was to assess the psychometric properties of the German language version of the SAQ.

**Methods:**

A survey was carried out in two University Hospitals in Switzerland in autumn 2009 where the SAQ was distributed to a sample of 406 nurses and physicians in medical and surgical wards. Following the American Educational Research Association guidelines, we tested the questionnaire validity by levels of evidence: content validity, internal structure and relations to other variables. Confirmatory factor analysis was used to examine factor structure. Cronbach’s alphas and inter-item correlations were calculated to examine internal consistency reliability.

**Results:**

A total of 319 questionnaires were completed representing an overall response rate of 78.6%. For three items, the item content validity index was <0.75. Confirmatory factor analysis showed acceptable model fit (RMSEA = 0.045; CFI = 0.944) for the six-factor model. Additional exploratory factor analysis could not identify a better factor model. SAQ factor scores showed positive correlations with the Safety Organizing Scale (r = .56 - .72). The SAQ German version showed moderate to strong internal consistency reliability indices (Cronbach alpha = .65 - .83).

**Conclusions:**

The German language version of the SAQ demonstrated acceptable to good psychometric properties and therefore shows promise to be a sound instrument to measure patient safety climate in Swiss hospital wards. However, the low item content validity and large number of missing responses for several items suggest that improvements and adaptations in translation are required for select items, especially within the perception of management scale. Following these revisions, psychometric properties should reassessed in a randomly selected sample and hospitals and departments prior to use in Swiss hospital settings.

## Background

Patient safety, defined as the avoidance, prevention, and amelioration of adverse events or injuries stemming from the processes of healthcare [1], has become a major focus of clinical care and research over the past two decades. The Institute of Medicine (IOM) 2000 report, “To Err is Human”, estimated that more than 98,000 patients in the USA die per year because of adverse events [2]. The IOM report triggered researchers to develop new systematic approaches to improve patient safety in health care environments [1,3]. Patient safety culture has an essential impact on the safety of health care environments and can be an essential pathway for the prevention of adverse events [4-6].

### Patient safety culture and measurement of patient safety climate

The British Health & Safety Commission defines safety culture as “the product of individual and group values, attitudes, perceptions, competencies, and patterns of behavior that determine the commitment to, and the style and proficiency of, an organization’s safety management” [7]. More specifically, patient safety culture is defined as a “subset of organizational culture, which relates specifically to the values and beliefs concerning patient safety within healthcare organizations” [8] and the term patient safety climate generally refers to the measurable components of safety culture such as management behaviors, safety systems, and employee perceptions of safety [9]. Although there are some conceptual differences between safety culture and safety climate, the two terms are often used interchangeable in the literature [10,11].

Two systematic reviews report the history, development and psychometric properties of a variety of instruments available to assess patient safety climate [6,12]. Out of the reviewed instruments, the Safety Attitudes Questionnaire (SAQ) is commonly used to assess healthcare workers' perceptions of patient safety related attitudes in various clinical areas and healthcare settings [5,13-17].

The SAQ is comprised of 30–60 items measured on a 5-point Likert scale [13]. Psychometric properties of this instrument have been analyzed and reported, showing evidence of validity and reliability. Sexton et al. [13] carried out six surveys of health care providers in the USA, UK and New Zealand to test the psychometric properties of the SAQ. Exploratory factor analysis yielded a six-factor solution with all item loadings higher than .50 on the corresponding factor and no item cross-loadings. Item-to-total correlations were moderate to strong (.73-.95) and showed higher correlations with corresponding factors than with the other factors. Further studies confirmed the instrument’s strong psychometric properties, with a Raykov’s rho of .90 and Cronbach’s alpha of .85 [5,13,17]. Results of confirmatory factor analysis were also strong, with RMSEA of 0.048 [5] and 0.030 [13] and CFI of 0.90 [13]. However, no German language version has been published. The purpose of this validation study was to explore the psychometric properties of a newly developed German language version of the SAQ. Hypotheses and research questions to rigorously check the validity and reliability of the SAQ are listed in Table 1.

**Table 1:** Research questions and hypotheses of the validation study

**Level of evidence and reliability**	**Number of research question (R) or hypothesis (H)**	**Research question or hypothesis**
Evidence based on content validity	R1	Are the items relevant and appropriate in terms of the patient safety climate construct?
	R2	Are the items clear and easy to understand?
Evidence based on internal structure	H1	The data from this study confirm the proposed six-factor model of the original SAQ.
	H2	Individual items of the SAQ show high correlations within its respective factor.
Evidence based on relationship to other variables	H3	There are moderate to strong correlations between the factor scores of the SAQ and scores on the Safety Organizing Scale.
Reliability: internal consistency	H4	SAQ shows good internal consistency.

## Methods

### Design, setting, sample

A cross-sectional survey was conducted in two Swiss University hospitals: The departments of orthopedic surgery (70 beds, 4 units) and cardiovascular surgery (60 beds, 5 units) of the Bern University hospital; and the department of internal medicine (180 beds, 5 units) of the Basel University hospital, respectively.

This study used a convenience sample of registered nurses (RN) and physicians (MD) (N = 406), working for at least one month in the participating departments, who had daily direct patient contact and were employed for at least 20% of the time. The sampling procedure was chosen because the designated hospital departments agreed to participate in the study since an academic service partnership was established between the two university hospitals and the Institute of Nursing Science of the University of Basel.

### Measures

#### Safety attitudes questionnaire

The SAQ is a modification of the Intensive Care Unit Management Attitudes Questionnaire (ICUMAQ) [4,18,19], which was originally derived from the Flight Management Attitudes Questionnaire [20]. It has been adapted for various clinical settings such as intensive care units, operating rooms, general inpatient wards and outpatient settings. The original extended version consists of 60 items including 30 core items that are identical in all clinical settings. The short form version includes only the 30 core items, four of which are responded to separately for the hospital and unit level, yielding a total of 34 items. Previous factor analysis identified factors covering six aspects of the safety climate: teamwork climate (6 items), job satisfaction (5 items), safety climate (7 items), stress recognition (4 items), working condition (4 items) and perception of management (4 items) [4,13,21]. Individuals respond to one of the working condition items and three of the perception of management items separately in relation to their unit and in relation to their hospital so there are a total of 34 possible responses. SAQ responses are given on a 5-point Likert scale (1 = disagree strongly, 2 = disagree slightly, 3 = neutral, 4 = agree slightly, 5 = agree strongly) including a "not applicable" option for each item. The values of two negatively worded items (Items 2,11) are reversed scored [22]. We decided to use the 30-item version of the SAQ because of its usability, good psychometric properties as shown in previous studies with Raykov’s rho of 0.90, CFI of 0.90 and RMSEA of 0.30 [13], and its broad implementation.

#### German version of the SAQ

The SAQ was translated from English to German and back again by native speakers following the adapted Brislin protocol [23]. The translated version was reviewed by a focus group consisting of faculty, nurse experts and physicians for clarity and appropriateness of wording and for each item’s meaning in the cultural setting of the German-speaking part of Switzerland.

#### Socio-demographic data

Demographic characteristics of participants were assessed using a structured questionnaire to obtain information on gender, profession (RN, MD), age (years), years working on the current unit and number of years working in their profession.

### Data collection

Data collection for the validation study occurred between September and November 2009.

The SAQ was distributed in 14 units of two Swiss University Hospitals during each unit’s team meeting. Reminders were sent to all participants after 14 days and again after 21 days to enhance the response rate. Questionnaires not returned after 24 days were considered non-responses.

### Statistical analysis

All data were analyzed using SPSS 15 (SPSS, Inc., Chicago, IL) except for factor analyses where Mplus version 7.1 (Muthén & Muthén, Los Angeles, CA) [24] was used. Descriptive statistics (means and standard deviations for normally distributed and interval scaled data as well as medians and interquartile ranges (IQR) for skewed interval scaled data and ordinal scaled data) were used to describe sample characteristics and missing values (MV). Mann Whitney U tests were used to verify MV pattern differences according to units and hospitals. All data were screened for outliers and normal distributions by considering boxplots and histograms. Statistical significance was set at < .05.

#### Psychometric testing of the German version of the SAQ

For the validity testing of the SAQ we followed the American Educational Research Association (AERA) standards for educational and psychological testing which describe the standard method for validity testing by five levels of evidence: 1) test content, 2) response processes, 3) internal structure, 4) relations to other variables, and 5) consequences of testing. In the current study, validity evidence was examined in relation to test content, internal structure and relations to other variables.

*Evidence based on test content* was examined to answer research questions 1 and 2 (Table 1). An interdisciplinary convenience expert group consisting of 16 nursing experts and physicians at the University Hospital in Basel was asked in 2009 to rate the relevance of each of SAQ item on a 4-point Likert-type scale, ranging from 1 (not relevant) to 4 (highly relevant). Accordingly, the item-content validity index (I-CVI) and scale-content validity index (S-CVI) were calculated based on the proportion of experts who gave a rating of 3 or 4. A S-CVI of more than 0.75 indicates good content validity [25].

*Evidence based on internal structure* of the SAQ was explored to test hypotheses 1 and 2 (Table 1). Literature shows different recommendations for an appropriate sample size for confirmatory factor analysis (CFA). The more recent suggestions are at least 10–15 subjects per item [26] and a minimal overall sample size of 300 cases, according to Tabachnick and Fidell [27]. CFA was carried out to verify the factor structure identified during testing of the original English language version of the SAQ (H1). CFA allows one to test whether a pre-hypothesized relationship can be confirmed between observed variables and their underlying latent dimensions. Confirmatory factor analyses were carried out using Mplus [28] using a robust weighted least squares approach (WLSMV) for estimation as the SAQ items are categorical and ordinal scaled with strong ceiling effects [28]. To ensure that the resulting bivariate tables for pairs of items had no zero cells, the five possible categories were collapsed into three categories: 1 = do not agree (combined responses for disagree strongly, disagree slightly, and neutral), 2 = agree slightly, and 3 = agree strongly. Items with missing data (which included missing and not applicable responses) for more than 11% of participants were excluded from the CFA. The decision to exclude items if more than 11% of participants had missing data was selected to be consistent with previous research where the missing data rate did not exceed 13% for any item [5,29]. The CFA was performed using available data from all 319 participants using pairwise present approach to handle missing data. We calculated the following indices through Mplus to assess goodness-of fit: comparative fit index (CFI) (should exceed 0.90 for an acceptable model fit [30,31]), the Tucker-Lewis index (TLI) (should be close to .95 for good model fit [30,31]), and the root mean square error of approximation (RMSEA) (should not exceed .06 for a good model fit [31]) and its 90% confidence interval. Additionally the traditional chi-square test statistics for the baseline model and model fit are reported. Modification indices (MI) were examined after the fitting of the CFA model to identify any additional adjustments. To check hypothesis 2, factor loadings of individual items were estimated based on the six-factor CFA model for the full sample.

*Evidence based on relations to other variables* was explored to check concurrent validity as expressed in hypothesis 3 (Table 1). The overall and subscale mean scores of SAQ were compared with those of a different questionnaire designed to measure patient safety climate, the Safety Organizing Scale (SOS). This instrument, developed by Vogues and Sutcliffe in 2006, is a 9-item index intended to measure the extent to which RNs and their colleagues engage in patient safety behaviors and practices on their clinical units. The SOS has excellent validity indices (CFI = .964, RMSEA = .055) and good internal consistency (Cronbach’s alpha = .88) [32]. The German version of the SOS showed good content validity indices (S-CVI >0.89) and good reliability indices (Cronbach’s alpha >0.79) [24]. The SOS was distributed together with the SAQ in the subgroup of all participating units of the University Hospital of Basel (N = 154). Spearman’s rank-order correlation was used to estimate the correlations between SAQ and SOS scores.

### Reliability of the SAQ

Internal consistency was examined to test hypothesis 4 (Table 1). Cronbach’s alpha was calculated for each factor of the SAQ. Values of Cronbach’s alpha exceeding 0.70 indicate adequate internal consistency [25,26]. Additionally, inter-item correlations and correlations between individual items and corresponding factor scores were calculated to examine the internal consistency reliability of SAQ (correlations greater than 0.30 indicate good reliability) [26].

### Ethical considerations

The study was approved by the ethics committees of the Cantons of Bern and Basel in June 2009.

## Results

### Demographics

A total of 319 questionnaires were completed by the clinicians representing an overall response rate of 78%. More specifically, 273 of 323 nurses (84%) and 46 of 83 physicians (55%) returned their questionnaires. On average, nurses worked on their current unit for 5 to 11 years (median), whereas physicians worked on their current unit for 1.0 to 2.5 years. Detailed socio-demographic and professional characteristics are shown in Table 2.

**Table 2:** Descriptive statistics of the study sample

	**Total sample**	**Inselspital Bern, University Hospital**		**University Hospital Basel**
		**Cardiovascular surgery**	**Orthopedic surgery**	**Internal medicine**
**Nurses**	N = 273	n = 69	n = 69	n = 135
Female (%)	86.7	92.3	91.2	90.5
Age (years), Median (IQR)	35 (20)	36 (22)	33 (15)	39 (20)
Number of years working				
In the unit, Median (IQR)	5 (11)	6 (7)	6.5 (6)	11 (14)
As nurse, Median (IQR)	9 (17)	10 (13)	8 (11)	14 (18)
**Physicians**	N = 46	n = 10	n = 17	n = 19
Female (%)	28.3	20	11.7	39.5
Age (years), Median (IQR)	34 (4)	32 (7)	35 (7)	33 (4)
Number of years working				
In the unit, Median (IQR)	1 (1.5)	1.6 (1.5)	2.5 (3)	1 (.75)
As physican, Median (IQR)	5.5 (4)	7.7 (9)	7.5 (5)	4.5 (4)

*IQR* = Interquartile range.

### SAQ response patterns

Missing values’ analysis showed no item with more than 2% missing values (range 0–1.9%).

However, analysis of “not applicable” responses revealed a much higher frequency for seven items (range 9.7%- 53.7%, see Table 3). These responses were treated as missing values (MV). Items asking participants about their perceptions of management and working conditions in relation to their hospital (23b, 24b, 25b, and 26b) were excluded from further analysis having MV’s > 25%. We found no significant differences in MV rates between departments (p = 0.544), hospitals (p = 0.827) or professions (p = 0.983). Additionally, results suggest that item 24 *“Management does not knowingly compromise the safety of patients”* was not clear at all to the participants. Its distribution showed a strongly bimodal pattern at both ends of the Likert scale and high rates of not applicable values. These results are confirmed by this item’s low CVI of .350. The item was therefore excluded from further analysis.

**Table 3:** SAQ item characteristics

**Dimensions and survey questions, numbered**	**% Missing data (i.e.,% answered not applicable)**	**Mean (SD)**	**Agree (%)**	**Disagree (%)**	CVI-Index*	CFA: Standardised model*	
						**Factor loading**	**S.E.**
**Teamwork climate**							
1. Nurse input is well received in this clinical area.	0 (0)	4.40 (.737)	88.4	1.3	0.81	0.660**	0.047
2. In this clinical area, it is difficult to speak up if I perceive a problem with patient care***.	0 (0.3)	4.33 (.984)	7.5	84.6	0.90	0.550**	0.056
3. Disagreements in this clinical area are resolved appropriately (i.e., not *who* is right, but *what* is best for the patient)	0.6 (1.3)	4.07 (.856)	77	4.2	0.78	0.626**	0.051
4. I have the support I need from other personnel to care for patients.	0.3 (0)	4.08 (.825)	80.5	3.5	0.88	0.572**	0.055
5. It is easy for personnel in this clinical area to ask questions when there is something that they do not understand.	0 (0)	4.50 (.726)	93.1	2.5	0.95	0.633**	0.052
6. The physicians and nurses here work together as a well-coordinated team.	0.3 (0)	3.65 (.786)	58.2	5.7	0.86	0.402**	0.062
**Safety climate**							
7. I would feel safe being treated here as a patient.	0 (0.6)	3.92 (.759)	72.2	2.5	0.95	0.610**	0.046
8. Medical errors are handled appropriately in this clinical area.	0 (2.8)	4.09 (.779)	81.6	3.9	0.88	0.707**	0.038
9. I know the proper channels to direct questions regarding patient safety in this clinical area.	0.9 (2.8)	4.42 (.725)	90.9	2.0	0.76	0.577**	0.055
10. I receive appropriate feedback about my performance.	0 (0)	3.77 (.990)	63.0	9.1	0.79	0.625**	0.043
11. In this clinical area, it is difficult to discuss errors***.	0 (0.3)	4.05 (.933)	7.2	79.2	0.95	0.609**	0.045
12. I am encouraged by my colleagues to report any patient safety concerns I may have	0 (2.5)	3.88 (.986)	70.4	10.0	0.86	0.550**	0.048
13. The culture in this clinical area makes it easy to learn from the errors of others.	0.3 (2.2)	3.93 (.973)	72.3	8.7	0.75	0.670**	0.041
**Job satisfaction**							
14. I like my job.	0 (0.3)	4.61 (.640)	94.3	0.9	0.75	0.602**	0.055
15. Working in this hospital is like being part of a large family.	0.6 (2.2)	3.62 (.954)	60.3	12.6	0.69	0.706**	0.047
16. This hospital is a good place to work.	0.3 (0.6)	4.28 (.745)	87.0	1.9	0.81	0.797**	0.033
17. I am proud to work at this hospital.	0.9 (1.6)	4.16 (.840)	83.0	4.2	0.73	0.790**	0.035
18. Moral in this clinical area is high.	0.3 (0.6)	4.05 (.781)	78.8	2.8	0.88	0.754**	0.038
**Stress recognition**							
19. When my workload becomes excessive, my performance is impaired.	0 (0.3)	3.47 (1.138)	53.0	21.8	0.88	0.757**	0.040
20. I am less effective at work when fatigued.	0.3 (0.3)	3.65 (1.108)	59.3	16.4	0.92	0.862**	0.032
21. I am more likely to make errors in tense or hostile situations.	0.3 (0.6)	3.62 (1.141)	59.2	20.3	0.86	0.793**	0.038
22. Fatigue impairs my performance during emergency situations (e.g., emergency resuscitation, seizure).	0.6 (9.7)	2.88 (1.327)	37.5	47.7	0.45	0.768**	0.048
**Perceptions of management**							
23. Management supports my daily efforts:							
Unit levels:	0.3 (0)	3.90 (.973)	67.5	7.5	0.81	0.556**	0.057
Hospital level:	0.6 (25.1)	2.80 (1.146)	31.6	39.7		Excluded from analysis	
24. Management does not knowingly compromise the safety of patients.							
Unit level:	0.9 (13.2)	3.42 (1.660)	59.1	34.7	0.35	Excluded from analysis	
Hospital level:	1.3 (33.5)	3.30 (1.448)	51.9	33.0		Excluded from analysis	
26. I am provided with adequate, timely information about events in the hospital that might affect my work.							
						
Unit level:	0.6 (2.6)	4.06 (.840)	77.6	4.9	0.78	0.676**	0.054
Hospital:	1.9 (26.0)	3.69 (.987)	66.5	13.0		Excluded from analysis	
27. The levels of staffing in this clinical area are sufficient to handle the number of patients.	0.3 (0)	3.45 (1.070)	49.1	15.7	0.82	0.345**	0.065
**Working conditions**							
25. Problem personnel are dealt constructively in hospital.							
						
Unit level:	0.9 (10.7)	3.71 (.995)	61.3	11.0	0.76	0.725**	0.064
Hospital level:	1.6 (53.7)	3.32 (.942)	39.9	16.2		Excluded from analysis	
28. This hospital does a good job of training new personnel.	0.6 (1.6)	4.03 (.969)	73.4	6.1	0.96	0.635**	0.049
29. All the necessary information for diagnostic and therapeutic decisions is routinely available to me.	0.6 (1.6)	4.14 (.719)	82.1	2.6	0.92	0.462**	0.062
30. Trainees in my discipline are adequately supervised.	0.3 (5.3)	4.24 (.830)	83.3	3.3	0.90	0.711**	0.049

*Analysis based on the 319 participants’ pairwise present data.

**p < .001 for all standardized factor loadings; ***Items reverse scored.

Distributions of all item responses were positively skewed in both hospitals. At the participant level, responses showed no floor effects but high ceiling effects (66-90%), i.e., there were participants who responded in 66-90% of the items with “agree strongly”. At the item level, there was a strong floor effect for item 22 *“Fatigue impairs my performance during emergency situations”* (42% of participants strongly disagreed with this item). On other items of the stress recognition factor the proportion of subjects who “strongly disagree” ranged from 15 to 20%. Ceiling effects were observed in five of the 34 items with 55 to 63% of participants responding that “agree strongly” with the statement. See Table 3 for descriptive data of the SAQ item characteristics.

### Content validity

Validity evidence based on content (research questions 1 and 2: Table 1) was evaluated by calculating scale and item level content validity indexes. The scale-level content validity index (S-CVI) was 0.83, indicating good content validity (research questions 1 and 2). For most of the items the I-CVI was also good, ranging between 0.78 and 0.95. However, four items “*Working in this hospital is like being part of a large family“, “I am proud to work at this hospital”, “Fatigue impairs my performance during emergency situations”*, and “*Management does not knowingly compromise the safety of patients*” showed an I-CVI below the recommended level of 0.75 (0.35-0.73). These items were discussed in the expert group and two of these items were retained because their I-CVIs were close to the recommended cut-off level and one was retained because it showed high loading to its respective factor “stress recognition”, a factor which arose in the analysis as a strong unitary factor, detached from all other factors. As aforementioned, the item “*Management does not knowingly compromise the safety of patients”*, was dropped out for further analysis because it was unclear to most participants. Content validity was not re-examined after this item was dropped.

### Internal structure

CFA based on the retained 29 items and using the full sample (N = 319) showed acceptable to good model fit (RMSEA = 0.045; CFI = 0.941, TLI = 0.937) (Table 4). The estimated loadings on the original factors were large and significant for all items, supporting hypothesis 2 (Table 3). Correlations between factors showed that all factors except for stress recognition are significantly correlated with each other, as expected for factors from the same scale and hypothesized in hypothesis 1. Nevertheless, the results of CFA highlighted some potentially problematic items in terms of factor allocation (all of them in the perceptions of management or working condition factors). The following three indices can be considered as an indicator of whether items can be mapped clearly to one factor (high values) or not (small values) [28]. Given these results of the CFA, we conducted an exploratory factor analysis (EFA) to explore if other factor structures could eliminate these problems. Residual variances were above 0.70 for items 25, 27, and 28; modification indices were large (>> usual cutoff of 3.84) for items 25, 27, 28, 30; and three items, 25, 27, and 28, had loadings of 0.40 or greater for more than three factors (i.e., multiple cross-loadings). Different models (e.g., Varimax, Geomin) showed good fit indices creating various factor structures (see Additional file 1). However, EFA identified many items with cross loadings and items with loadings below 0.40 for all factors. No better factor structure than in the original SAQ could be found.

**Table 4:** Results of confirmatory factor analysis (CFA)

Goodness of fit index*	Model using full sample** (N = 319)
Chi-square Test of Model Fit (df, p-value)	598.559 (362, <.001)
Chi-square Test of Model Fit for the Baseline Model	4405.693
(df, p-value)	(406, <.001)
Comparative Fit Index (CFI)	0.941
Tucker-Lewis-Index (TLI)	0.934
Root Mean Square Error of Approximation (RMSEA)	0.045
90% CI for RMSEA	0.039, 0.052
Probability RMSEA ≤ .05	0.887

*Calculated using the robust weighted least squares (WLSMV) estimation method.**Missing data are handled using a pairwise present approach.

### Relations to other variables

Evidence based on relations to other variables was tested by examining the relationship between SAQ and the Safety Organizing Scale. As hypothesized (H3), SAQ factor scores showed strong positive correlations with the total SOS score (teamwork climate: r = .579, safety climate: r = .619; job satisfaction: r = .575; stress recognition: r = .017; perception of management: r = .591; working climate: r = .554) except for stress recognition.

### Reliability of the hypothesized factor model

As predicted in hypothesis 4, the hypothesized factor model showed good reliability values. Cronbach’s alpha for the various factors varied from .436 to .791 (Table 5).

**Table 5:** Reliability characteristics of the six factors

**Factors of SAQ**	**Cronbach’s alpha**	**Inter-item correlation**	**Item to factor correlation, range**
Teamwork climate	.647	.241	.309-.406
Safety climate	.754	.280	.389-.567
Job satisfaction	.791	.435	.464-.677
Stress recognition	.789	.490	.546-.661
Perception of management	.436	.222	.163-.413
Working conditions	.647	.320	.172-.369

## Discussion

This study evaluated the psychometric properties of the SAQ German-language version. The results showed moderate to strong reliability, good content validity and acceptable CFA fit statistics. However, there were a few items, especially in the perception of management factor (Table 6) that were problematic and require additional investigation and refinement.

**Table 6:** Items with need for adjustments

**Item**	**Item statement**	**Reason for adjustment**	**Recommended improvements**
15	Working in this hospital is like being part of a large family.	Low CVI	Translational adaptations needed because of cultural distinctions
17	I am proud to work at this hospital	Low CVI	
22	Fatigue impairs my performance during emergency situations (e.g., emergency resuscitation, seizure).	Low CVI, high percentage of MV	
24a&b	Management does not knowingly compromise the safety of patients. Hospital and unit level	Low CVI, high percentage of MV, bimodal distribution	Translational adaptation needed because of problems in comprehensibility and clearness.
23b	Management supports my daily efforts. Hospital level only	high percentage of MV	Specification of the term “hospital level” needed
25b	Problem personnel are dealt constructively in hospital. Hospital level only	high percentage of MV	
26b	I am provided with adequate**,** timely information about events in the hospital that might affect my work. Hospital level only	high percentage of MV	
27, 28	27: The levels of staffing in this clinical area are sufficient to handle the number of patients	Cross loadings and high modification index in CFA	Problem known from other studies. Allocation to other factors is possible.
	28: This hospital does a good job of training new personnel		

*CVI* Content validity Index, *MV* Missing Values, *CFA* Confirmatory factor analysis.

### Content validity and missing values

Four items were isolated as potentially problematic through the content validity index. In terms of the internal structure of the SAQ’s German language version, our results indicate the need for additional investigations after refinement of the problematic items. Despite this, the preponderance of the evidence from reliability, content validity, and the factor structure suggest that this translation is reasonably acceptable. This is not unlike; similar results from previous studies of the SAQ’s psychometric properties, demonstrating good model validity and reliability [5,13,17,21,33]. Compared to other studies, the response rate in our study was high for both nurses (84%) and physicians (55%). In accordance with Cook, Dickinson, and Eccles [34], this can be explained by the successful implementation of a reminder system, whereby personally addressed notes were distributed to all participants in weeks two and three of data collection.

However, seven of the 34 items showed high missing value rates (9.7% to 53.6%, primarily due to “not applicable” responses). Previous studies had no items whose missing values exceeded 13% [5,13,29,33]. Most MVs were on items related to the perception of management factor with items focusing on hospital management having far more MVs than those focusing on unit management. This finding is quite different from US studies, but similar to those of Scandinavian studies. A possible explanation for this could lie in the hierarchical structure and culture in European hospitals, especially in German speaking parts of Europe. This may indicate that hospital management is further away from our Swiss sample of caregivers and may, therefore, be more difficult for them to comment on. This conjecture is strengthened by the fact that we could find no differences regarding MVs between hospitals, units or professions. It seems important, to consider these cultural differences when translating questions about hospital management. Furthermore, 9.7% of the participants had missing or not applicable responses to the item *“Fatigue impairs my performance during emergency situations”*. This suggests that this item is difficult to translate from English and difficult for some participants to understand. Our finding, are consistent with those reported in the Norwegian study [5] where this item was also unclear to some participants. The low I-CVI and bimodal distribution of responses may be further indicators of translation related problems [25]. Four items (15, 17, 22, and 24 Table 3) showed I-CVI values below 0.75. These low I-CVIs, especially together with the many MVs for some items, suggest that participants may have had difficulty understanding some of the items and that their wording needs further improvement.

### Commenting on the factor analyses

When we performed the CFA, we dropped one item based on its low I-CVI and its bimodal respons pattern and four items asking participants about their perceptions of management and working conditions in relation to their hospital based on their non-applicable- responses exceeding 25%. Given that our CFA was conducted on a smaller pool of items than the original CFA, caution should be exercised when comparing our findings to those of the original factor analysis [13]. The result of our CFA needs to be considered from two different point of views: The validity indices demonstrated acceptable model fit, implicating the confirmation of the hypothesized factor structure, as shown in other studies as well [5,21]. However, when considering the factor allocation indices in EFA (modification index, residual variance, cross loadings), results showed low values for items 25, 27, 28, and 30. All these items were part of either the perception of management or the working condition factor. Our results indicated that the allocation of these items to one factor seems to be not definite. Other studies support these findings reporting that item 27 and 28 sometimes loaded on different factors [5,21]. Translational as well as cultural differences may be the reason for the high modification indices for some of the items. Item 25 about “*problem personnel*” showed moderate loadings for all factors and did not clearly load on any one factor. This finding is not consistent with previous studies. This may be related to language and organizational cultural differences in the study sites.

### Additional reflections on the SAQ German language version

First, we must explore the role of the cultural setting on item responses. Two factors - stress recognition and job satisfaction - yielded factor structure in EFA and CFA identical to those for American and Northern Europe respondents, suggesting a common meaning for all participants, independent of their cultural setting [5,13,17,33,35]. The Norwegian study’s authors suggested that the question about staffing level related more to working conditions than to perceptions of management, while the reverse could be said for the item dealing with problematic personnel [5]. Our results confirmed these suggestions, considering that the modification indices for these questions are high. Perhaps staffing levels directly influence European caregivers’ perceptions of their working conditions. Second, as explained above, some of our results point to translational problems. Other authors reported similar problems which may be corrected by translational improvements [5]. Therefore it will be important to work more on the wording of some items including systematic cognitive testing. Third, we had to collapse the response options in order to meet CFA criteria. For the current study, the original Likert scale response categories 1 = disagree strongly, 2 = disagree slightly, and 3 = neutral were collapsed into a single value which were labeled as did not agree [22]. The high and significant factor loadings for 5 of the 6 previously identified factors suggests that with minimal translational improvements and cultural adaptations (targeting especially the perception of management scale), the SAQ German version may fit the original factor structure even better, a hypothesis which will be tested in future studies.

### Limitations

An important limitation to this study was the high percentage of missing and not applicable values for several items, especially those items where the hospital was the frame of reference, which necessitated the omission of these items from analyses. Another limitation of this study was the very low response rates for the disagree strongly and disagree slightly categories for many items. To avoid zero cells in bivariate tables, the disagree strongly, disagree slightly, and neutral categories were collapsed into a single category resulting in three ordinal response categories (did not agree/neutral, agree slightly and agree strongly) instead of the original 5 categories. This may have contributed to the difficulties we identified with factor loading for some items. With collapsing of adjacent ordered categories, there is loss of item information which may affect the magnitude of the correlation coefficients between items, the data elements utilized in factor analysis. Had we been able to utilize all response choices, the findings may have been different. These different findings would arise rather because of the cultural characteristics of the included organizations which could be distinguished in a better way when having all response choices, than because of the augmented size of the sample. In addition, based on our sample size we were slightly underpowered and this, along with missing values and the distribution pattern with ceiling effects may have contributed to our moderate results in terms of the internal structure. Also, as we used a small convenience sample for this study, the results are not generalizable.

### Recommendations

The German language version of the SAQ demonstrated acceptable to good psychometric properties and therefore shows promise to be a sound instrument to measure patient safety climate in Swiss hospital wards. The results of this study, however, suggest that revisions need to be made to some of the items and that some of the items may not be relevant in the Swiss hospital culture. We recommend that future studies utilize focus group methodology (including both frontline staff and clinical experts) to gather qualitative information that can be utilized to modify problematic items in terms of content and/or language. Following these adaptations, the psychometric properties need to be reassessed in a randomly selected sample of hospitals and departments.

## Conclusions

The current German version of the SAQ appears to demonstrate reasonable psychometric properties, but the perceptions of management factor requires further improvements and adaptations in translation before being used in Swiss hospital settings. This study should be considered the initial step to establish the psychometric properties of the German language version of the SAQ. Additional research is needed to refine the instrument and to re-examine its psychometric properties based on those refinements.

## Competing interests

The authors declare that they have no competing interests.

## Authors’ contributions

NZ has developed the study design, conducted the research, carried out the descriptive analyses, participated in the interpretation of the study results, drafted and revised the manuscript. KK participated in the interpretation of results, reviewed and contributed to the manuscript writing. SS and SE executed the confirmatory factor analysis and extended explanatory factors analysis, provided support in the interpretation of the CFA-results and reviewed and contributed to the manuscript writing and revisions. BS was the provided support in interpretation of results, reviewed and contributed to the manuscript writing. RS was the study mentor. He developed the research conception, provided continuous support in view of data analysis, interpretation of results, reviewed and contributed to the manuscript writing and revisions. All authors read and approved the final manuscript.

## Pre-publication history

The pre-publication history for this paper can be accessed here:

http://www.biomedcentral.com/1472-6963/13/347/prepub

## Supplementary Material

Additional file 1 Summary of exploratory factor models with acceptable fit indexes. Click here for file
