# Supplementary material for: Assessing the safety attitudes questionnaire (SAQ), German language version in Swiss university hospitals - a validation study
Source: BMC Health Serv Res. 2013 Sep 10;13:347. doi: 10.1186/1472-6963-13-347 (PMC3846625; doi:10.1186/1472-6963-13-347)
Supplement: Additional file 1 — Summary of exploratory factor models with acceptable fit indexes. [file 1472-6963-13-347-S1.doc]

| Model  **Additional file 1: Summary of exploratory factor models with acceptable fit indexes** | # Factors | X^2^  Should be ns | RMSE  Should be <0.06 | TLI  Should be close to 0.95 | CFI  Should be > 0.90 | # cross loadings  (two values >.40) | > 2 items without values ≥ .40 |
| --- | --- | --- | --- | --- | --- | --- | --- |
| Varimax,  Full sample (N=319) | 6  7  8  9 | 300.58, p=.01  265.75, p=.03  237.50, p=.04  200.52, p=.13 | 0.026  0.024  0.023  0.018 | 0.978  0.98  0.98  0.99 | 0.987  0.99  0.98  0.99 | 1, 7, 8, 13  1, 8, 13  8, 13  1, 8,13 | 9, 10, 12  9, 10, 27  10, 27  10, 24A |
| Varimax,  Partial sample  (N=194) | 5  6  7 | 311.68, p=.45  276.24, p=.10  237.77, p=.25 | 0.028  0.025  0.018 | 0.975  0.98  0.99 | 0.98  0.99  0.99 | 3, 5, 7, 8, 26A  3, 5, 7, 8, 30  8, | 9, 12, 29  10, 12, 24A, 29  10, 12, 23A |
| Geomin,  Full sample  (N=319) | 6  7  8  9 | 300.58, p=.01  265.75, p=.03  237.40, p=.04  200.52, p=.15 | 0.026  0.024  0.023  0.018 | 0.98  0.98  0.98  0.99 | 0.99  0.99  0.99  0.99 | 12  3  3, 8 | 7, 9, 12, 13, 27  9, 10, 27  10, 27  7, 10 |
| Geomin,  Partial sample  (N=194) | 5  6 | 311.68, p=.05  276.24, p=.10 | 0.028  0.025 | 0.98  0.98 | 9.98  0.99 | 28  26A | 12, 13, 27  10, 12, 23A, 29 |
| Quartimin,  Full sample  (N=319) | 6  7  8  9 | 300.58, p=.01  265.75, p=.03  237.50, p=.04  200.52, p=.15 | 0.026  0.024  0.023  0.018 | 0.98  0.98  0.98  0.99 | 0.99  0.99  0.99  0.99 |  | 3, 7, 9, 10, 12, 25A, 27  3, 9, 10, 13, 27  10, 13, 26A, 27  1, 7, 10, 12, 13, 23A, 29 |
| Quartimin,  Partial sample  (N=194) | 5  6 | 319.68, p=.045  276.24, p=.10 | 0.028  0.025 | 0.97  0.98 | 0.98  0.99 |  | 8, 9, 10, 12, 13, 23A, 27  7, 8, 10, 12, 23A, 27 |
